# Cornelia De-Lange Syndrome: A Case Report

**DOI:** 10.5005/jp-journals-10005-1201

**Published:** 2013-08-26

**Authors:** Diana Noshir Mehta, Rupinder Bhatia

**Affiliations:** Assistant Professor, Department of Pediatric and Preventive Dentistry Government Dental College, Mumbai, Maharashtra, India, e-mail: drdiananmehta@gmail.com; Professor and Head, Department of Pediatric and Preventive Dentistry Pad. Dr. DY Patil Dental College and Hospital, Navi Mumbai Maharashtra, India

**Keywords:** Cornelia de-Lange syndrome, Craniofacial, Diagnosis

## Abstract

Cornelia de-Lange syndrome is a congenital anomaly syndrome characterized by distinctive facial dysmorphism, primordial short stature, hirsutism, and upper limb reduction defects that range from subtle phalangeal abnormalities to oligodactyly. Craniofacial features include synophrys, arched eyebrows, long eyelashes, small widely spaced teeth and microcephaly. IQ ranges from between 30 and 102 with an average of 53. Many individuals demonstrate autistic and self-destructive tendencies. It is an autosomal dominant disorder caused by specific gene mutations and occurrence is one in 30,000 to 50,000 children. This article describes a report of a classical case of the syndrome of a 10-year-old boy and emphasizes the oral and systemic findings. The role of the pediatric dentist, with his expertize in prevention, skills of behavior management and timely referral to medical speciality, is of paramount importance in the management of children with this syndrome.

**How to cite this article:** Mehta DN, Bhatia R. Cornelia De-Lange Syndrome: A Case Report. Int J Clin Pediatr Dent 2013;6(2):115-118.

## INTRODUCTION

Cornelia de-Lange syndrome (CdLS) was first described as a distinct syndrome in 1933, by Dr Cornelia de-Lange, a Dutch pediatrician, after whom the disorder has been named, though the first ever documented case was in1916 by Dr Brachmann.^1^ A gene responsible for CdLS–NIPBL on chromosome 5–was discovered in 2004 by researchers at Children's Hospital of Philadelphia. In 2006, a second gene–SMC1A on the X chromosome–was found by Italian scientists. A third gene discovery was announced in 2007.^2^ The gene SMC3 is on chromosome 10 and was also discovered by the research team in Philadelphia. The latter two genes seem to correlate with a milder form of the syndrome. The vast majority of cases are due to spontaneous mutations, although the defected gene can be inherited from either parent, making it autosomal dominant. The types of mutations seen in CdLS rarely include large deletions and 50% have detectable point mutations (frame shift, splice site, nonsense and missense).^3^

Most of the signs and symptoms of CdLS may be recognized at birth or even prenatally by ultrasound imaging. The incidence is 1 case per 10,000 to 50,000 births.^1^ No difference based on race and sex has been reported. Most children could not live more than 2 years and the main cause of death was pneumonia along with cardiac, respiratory and gastrointestinal abnormalities.^2^

Currently diagnosis is made on the basis of clinical observations.^4^ A thorough medical evaluation including a history and physical examination, family history, laboratory tests, X-rays and chromosome analysis is usually conducted before a diagnosis is made. DNA testing is helpful for confirmation of a clinical diagnosis, but the sensitivity is only 50% for mutations in NIPBL. There is the potential for CdLS to be caused by other genes which have yet to be identified.

CdLS has been characterized by retardation in growth, distinctive facial dysmorphism, primordial short stature, psychomotor delay, behavioral problems, hirsutism and upper limb reduction defects that range from subtle phalangeal abnormalities to oligodactyly.^5^

Reports of craniofacial features of CdLS include microbrachycephaly, synophrys, arched eyebrows, long eyelashes, depressed nasal bridge, anteverted nares, long philtrum, thin upper lip, high arched palate, late eruption of small widely spaced teeth, micrognathia, spurs in the anterior angle of mandible and prominent symphysis.^6^

In this paper, we report the various oral and clinical features along with radiographic and other investigations carried out for better management and treatment of children with this rare syndrome.

## CASE REPORT

**Fig. 1:**
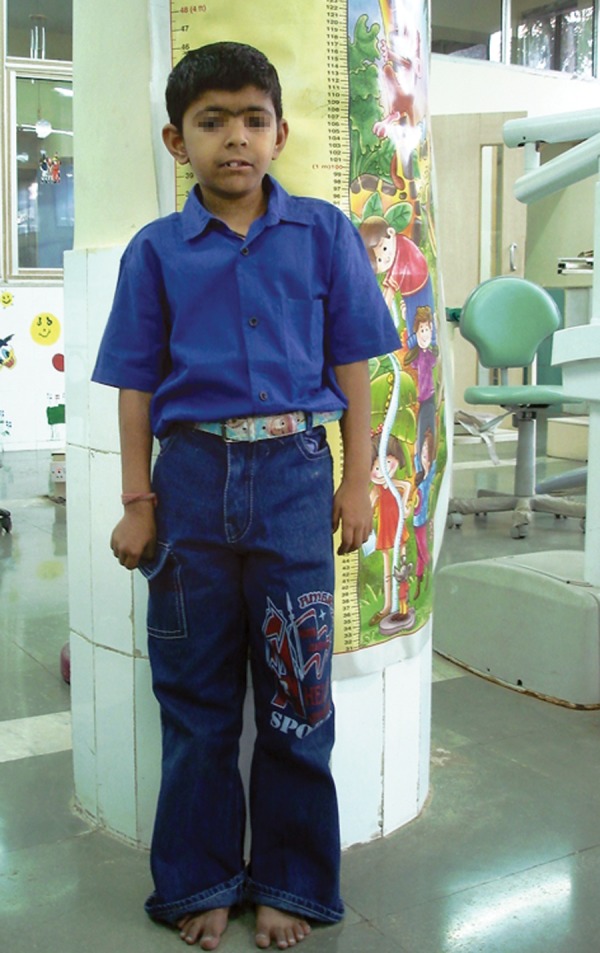
A 10-year-old male patient with Cornelia de Lange's syndrome

A 10-year-old male patient (Fig. 1) with CdLS, who demonstrated the classic facial features of the syndrome, reported with a chief complaint of decayed teeth in both upper and lower jaws. Past dental history showed that the patient had a history of swelling over right submandibular region associated with right lower back teeth (84, 85) 2 months back. History revealed that the child had low birth weight (1.75 kg), birth asphyxia, still birth, history of convulsions at 7 months, grossly delayed milestones and also demonstrated autistic and self-destructive tendencies.

## CLINICAL AND SYSTEMIC EXAMINATION

### Growth

Retarded osseous maturation.

### Development

Mental retardation, grossly delayed milestones, initial hypertonicity, low pitched, weak, growling, cry in infancy.

### Craniofacial (Fig. 2)

*Cranium:* Microbrachycephaly.

*Eyes:* Bushy eyebrows and synophrys, long, curly eyelashes, history of epiphora of left eye till age of 5 years, nystagmus.

*Nose:* Depressed nasal bridge**,** anteverted nares.

*Mouth:* Long philtrum, thin upper lip, and downturned angles of mouth, high-arched palate, delayed eruption, crowding of teeth in maxillary arch.

*Mandible:* Micrognathia***,*** spurs in the anterior angle of the mandible.

### Rest of Body

*Skin:* Hirsutism**,** Cutis marmorata, hypoplastic nipples and umbilicus.

*Hands and arms:* Micromelia, clinodactyly of fifth fingers, simian crease (Fig. 3).

*Feet:* Micromelia (Fig. 4).

**Fig. 2:**
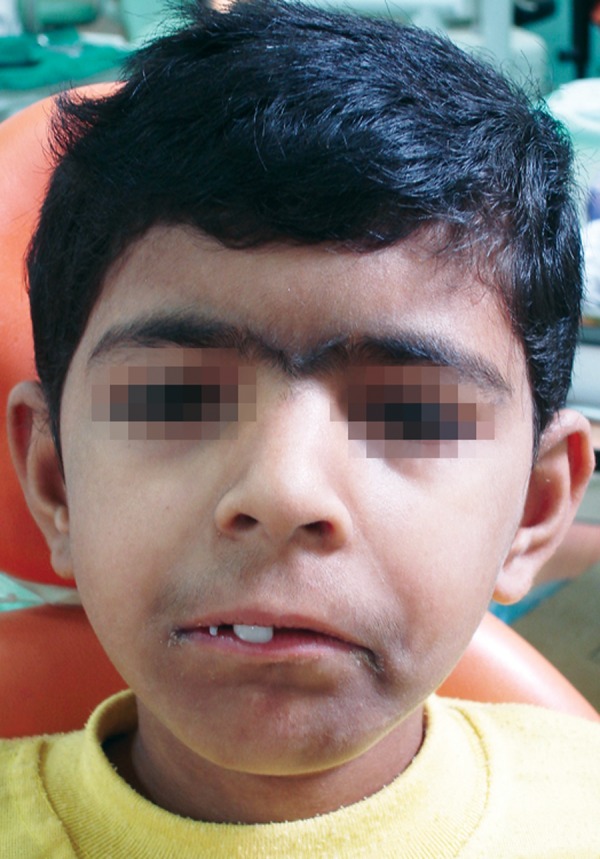
Classic craniofacial features of the syndrome

**Fig. 3:**
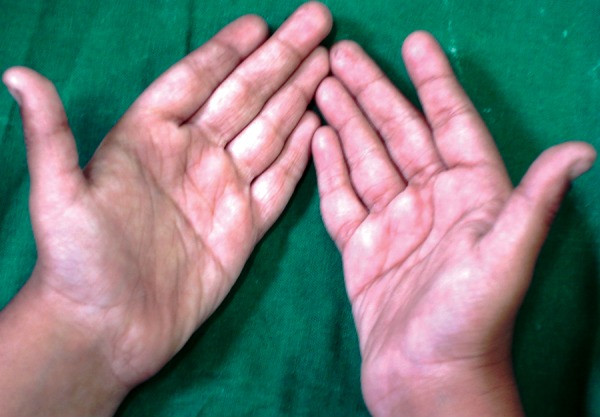
Micromelia, clinodactyly of fifth fingers, simian crease seen on the hands

**Fig. 4:**
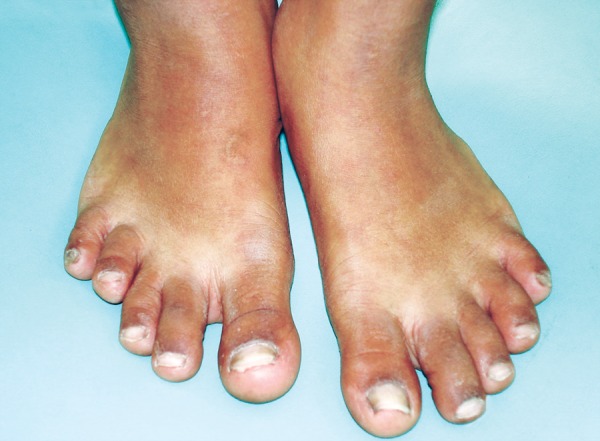
Micromelia seen of the feet

*Male genitalia:* Hypoplasia, undescended testes, cryptorchidism with atrophic testes.

*Other:* Myopia, ptosis and nystagmus, low posterior hairline, short neck, gastroesophageal reflux, partial hearing loss, seizure disorder.

## RADIOGRAPHIC FINDINGS

### Hand-wrist Radiographs (Fig. 5)

Proximal row of carpal bones is absent on both sides, clinodactyly of fifth finger is seen more on left side, first metacarpal is short on both sides, epiphysis at the lower end of ulna appears small and hypoplastic.

### Elbow Radiographs (Fig. 6)

Epiphysis at upper end of radius is small. At 10 years of age the patient had:

- Weight of 20 kg
- Height of 115.5 cm
- Head circumference of 48 cm
- IQ of 20 to 30 showing profound mental retardation.

**Fig. 5:**
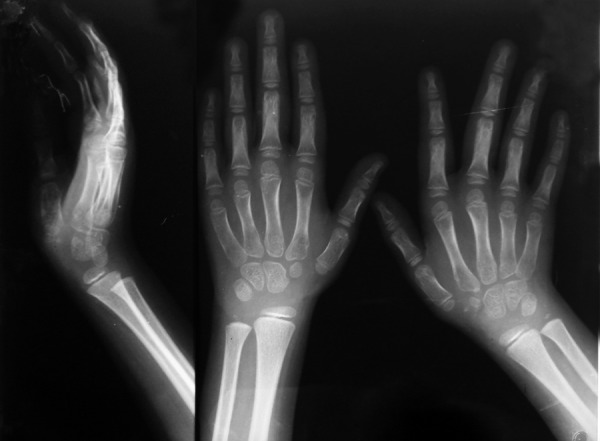
Hand-wrist radiographs

**Fig. 6:**
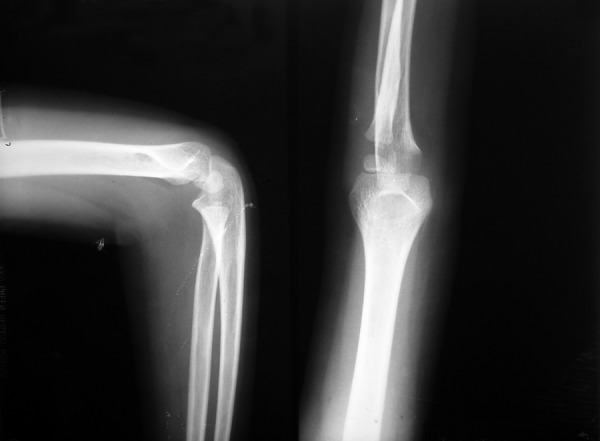
Elbow radiographs

**Fig. 7:**
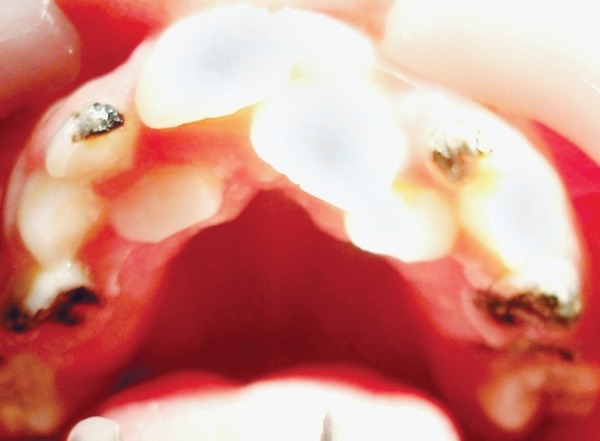
Occlusal view of the maxillary arch

**Fig. 8:**
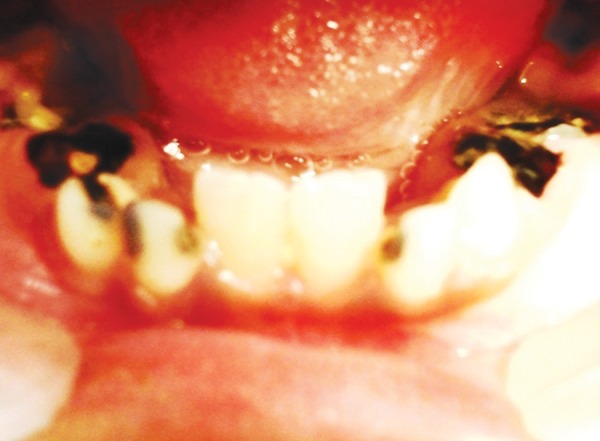
Occlusal view of the mandibular arch

Dental findings were as follows (Fig. 7 and 8)

- Teeth present:16 55 54 13 52 12 11 21 22 62 23 64 65 26 46 85 84 83 82 41 31 72 73 74 75 36
- Grossly carious: 54, 55, 64, 65, 74, 75, 84, 85
- Grade 2 mobile: 72, 82
- Over retained: 52, 62
- Class I caries with 36, 46 and class V caries with 83.

A comprehensive dental treatment was planned and all grossly carious root pieces, over retained and grade II mobile teeth were extracted. Class V cavity preparation on 83 was done and restored with GIC and class I cavity preparation on 36 and 46 were restored with silver amalgam restoration. Oral prophylaxis and fluoride application was done and regular 6 months follow-up was scheduled.

## DISCUSSION

CdLS is a congenital anomaly syndrome characterized by distinctive facial dysmorphism, primordial short stature, hirsutism, and upper limb reduction defects, distinct craniofacial features and low IQ ranges. Many individuals demonstrate autistic and self-destructive tendencies.^7^Currently, diagnosis is made on the basis of clinical observations. The case reported here closely confirms to the classical picture of CdLS.

The role of the pediatric dentist is to use appropriate skills of behavior management and his expertize in preventive dentistry by adopting the use of fluorides, sealants, stringent oral hygiene practices and providing timely diet counseling.^8^ The treatment of specific dental problems like erosion, gingival and periodontal disease, caries and jaw-size tooth material discrepancies should be dealt with appropriately. Also, timely referral to the medical speciality including the geneticist, cardiologist, gastroenterologist, endocrinologist, nephrologists, ophthalmologist, ENT specialist, speech therapist and occupational therapist should be met with as required.^6^ Beck, discussed the postmortem examination of the patients and revealed various congenital malformations of internal organs including cardiac defects, pulmonary hypoplasia, diaphragmatic hernias, gastrointestinal and genitourinary anomalies.^7^ Van Alien demonstrated ectopic neurons in cerebral white matter in new born and microcytic changes in the kidney of some patients.^9^ In the present case, various oral and clinical features along with radiographic and other investigations closely evaluated for better management and treatment of children with this rare syndrome. Dental treatment was planned in the present case and carried out as required. A regular follow-up was planned and emphasis on preventive dentistry was laid.

Life expectancy is normal if no major malformations like apnea, cardiac and gastrointestinal complications occur. Differential diagnosis includes Fryns syndrome and fetal alcohol syndrome which should be ruled out.^10^

The children with CdLS usually have a wide array of health problems, making it important for all specialists to be aware of the child's special needs. Multidisciplinary treatment approach is the key to success in managing children with syndromes. The pediatric dentist may be the first health personnel to identify such a child and may lead the multidisciplinary team in treating their problems.
